# Early Emergence of *Dickeya solani* Revealed by Analysis of *Dickeya* Diversity of Potato Blackleg and Soft Rot Causing Pathogens in Switzerland

**DOI:** 10.3390/microorganisms9061187

**Published:** 2021-05-31

**Authors:** Jacques Pédron, Santiago Schaerer, Isabelle Kellenberger, Frédérique Van Gijsegem

**Affiliations:** 1 Institute of Ecology and Environmental Sciences-Paris, Sorbonne Université, INRAE, 4 Place Jussieu, F-75252 Paris, France; jacques.pedron@upmc.fr; 2 Agroscope Changins, Domaine de Recherche Protection des Végétaux, CH-1260 Nyon, Switzerland; santiago.schaerer@agroscope.admin.ch (S.S.); isabelle.kellenberger@agroscope.admin.ch (I.K.)

**Keywords:** comparative genomics, blackleg, soft rot Pectobacteriaceae, Swiss Agroscope collection

## Abstract

Blackleg and soft rot in potato caused by *Pectobacterium* and *Dickeya* enterobacteral genera are among the most destructive bacterial diseases in this crop worldwide. In Europe, over the last century, *Pectobacterium* spp. were the predominant causal agents of these diseases. As for *Dickeya*, before the large outbreak caused by *D. solani* in the 2000s, only *D. dianthicola* was isolated in Europe. The population dynamics of potato blackleg causing soft rot *Pectobacteriaceae* was, however, different in Switzerland as compared to that in other European countries with a high incidence (60 up to 90%) of *Dickeya* species (at the time called *Erwinia chrysanthemi*) already in the 1980s. To pinpoint what may underlie this Swiss peculiarity, we analysed the diversity present in the *E. chrysanthemi* Agroscope collection gathering potato isolates from 1985 to 2000s. Like elsewhere in Europe during this period, the majority of Swiss isolates belonged to *D. dianthicola*. However, we also identified a few isolates, such as *D. chrysanthemi* and *D. oryzeae*, two species that have not yet been reported in potatoes in Europe. Interestingly, this study allowed the characterisation of two “early” *D. solani* isolated in the 1990s. Genomic comparison between these early *D. solani* strains and strains isolated later during the large outbreak in the 2000s in Europe revealed only a few SNP and gene content differences, none of them affecting genes known to be important for virulence.

## 1. Introduction

Potatoes are cultivated all over the world, as far north as Finland near the Arctic Circle and far south as New Zealand, and in various environmental conditions from the South American Altiplano to the Neguev Desert in Israel; it is the fourth main food crop worldwide. In seed potato production, besides bacterial wilt caused by *Ralstonia solanacearum* and, to a lesser degree, bacterial ring rot and wilt caused by *Clavibacter sepedonicus*, the most destructive bacterial diseases are blackleg and soft rot provoked by the genera *Pectobacterium* and *Dickeya* [1]. In several European countries, these soft rot *Pectobacteriaceae* (SRP) are responsible for most of the declassifications and rejections of potato seed lots [2]. The main route of infection is via contaminated potato seeds, where the bacteria may remain latently at a low level until the environmental conditions become favourable for expression of the virulence factors and extensive bacterial multiplication. The most important factor is the production and secretion of a battery of plant cell wall degrading enzymes that allow the maceration of plant tissues, leading to cell lysis and liberation of the cell content. However, SRP virulence relies on several other factors that allow these bacteria to adapt to environmental changes encountered *in planta* and to face the stresses produced by plant defence responses [3,4,5].

The population dynamics of the SRP causing blackleg and tuber rot in potato is well described in Europe. Historically, in most European countries, the causal agent of potato blackleg and soft rot was the cold-tolerant *P. atrosepticum* [6]. The first report of *Dickeya* (at the time called *Erwinia chrysanthemi*) recovered from potatoes dates from the 1970s, but losses attributable to this pathogen have remained generally low and sporadic [7]. In the 1980–1990s, surveys performed at the NAK in The Netherlands showed a more complex situation with a greater incidence of *Dickeya* (around 20%) and the occurrence of members of the so-called *Pectobacterium carotovorum* complex (at the time called *Erwinia carotovora*) (E. G. de Haan, NAK, personal communication). Analysis of *Erwinia chrysanthemi* strains isolated from potatoes in Europe during this period [8,9] appeared to be mostly *D. dianthicola*, a species first isolated in ornamentals in the late 1950s in UK and Denmark [10]. From 2005 to 2013, a large outbreak in seed potatoes ravaged Europe. It was accompanied by a high incidence of *Dickeya* that reached, for example, up to 70% in The Netherlands (E. G. de Haan, NAK, personal communication). From 2014, *Dickeya* was progressively replaced by *P. brasiliense* that now is isolated from more than 80% of blackleg-suffering plants (E. G. de Haan, NAK, personal communication).

The 2005 outbreak coincided with the isolation of a new *Dickeya* species, *D. solani*, which has commonly been isolated from seed potato tubers in The Netherlands—even though a wide range of different cultivars and locations have been sampled—but has also appeared to predominate in other European countries and Israel [7,11,12,13].

The population dynamics was different in Switzerland since *Dickeya* spp. have been described as being the cause of blackleg in 60–90% of tested diseased potato plants since the 1980s [14,15]. Thus, we took advantage of the constitution in the Swiss Agroscope of a collection of former *Erwinia chrysantemi* since the 1980s to analyse the history of *Dickeya* diversity in potatoes in this particular case.

## 2. Material and Methods

### 2.1. Bacterial Strains, Culture Conditions, DNA Extraction and Species Identification

The bacterial strains used in this study are presented in Table 1. They were isolated from diseased stems belonging to potato plants eliciting black leg symptoms in the multiplication fields or from rotted potato tubers. Stems or tubers were washed under running tap water, blotted dry with paper towels and cut open longitudinally. Small pieces (20–40 mg) of tissue were removed at the edge of the rotting lesions, close to healthy tissue. Those pieces were further shredded in 2 mL of sterile distilled water and left to macerate for 10 min. Aliquots from such suspensions were streaked onto crystal violet pectate (CVP) medium and tested for cavity formation following incubation at 27 °C for 24–48 h. Bacteria were re-isolated from characteristic pits, plated onto KB medium, and incubated at 27 °C for 24–48 h. Colonies were further characterised by biochemical tests [14,15]. They were routinely grown on LB^-^ medium consisting of LB medium without added NaCl (10 g/L peptone, 5 g/L yeast extract). 

Total bacterial DNA was extracted using the Wizard genomic DNA purification kit (Promega) following manufacturer’s protocol, and determination of appurtenance to *Dickeya* species was determined by PCR amplification of the housekeeping gene *gapA* using the gapA-7-F and gapA-938-R primer set and Sanger sequencing of the *gapA* amplicon [16] using the Eurofins Custom DNA Sequencing PlateSeq Service. The phylogenetic tree was reconstructed from the *gapA* nucleotide sequences. The gapA genes were aligned using MUSCLE [17] software and were filtered using the GBLOCK tool [18]. The alignments were used for building a phylogenetic tree with the PhyML algorithm based on the Tamura–Nei model [19] with SeaView software [20], with 200 bootstrap replications.

### 2.2. Genome Sequencing and Assembly

To analyse the diversity of the early *D. solani* strains, genome sequencing was performed at the next-generation sequencing core facilities of the Institute for Integrative Biology of the Cell (Avenue de la Terrasse, 91190 Gif-sur-Yvette, France). Nextera DNA libraries were prepared from 50 ng of high-quality genomic DNA. Paired-end 2 × 75 pb sequencing was performed on an Illumina NextSeq500 apparatus, with a High Output 150 cycle kit. CLC Genomics Workbench (Version 9.5.2, Qiagen Bioinformatics, Hilden, Germany) was used to assemble reads. Final sequencing coverage was between 49 and 79 (Table 1). Coding sequences were predicted using the RAST server [21] with the Glimmer 3 prediction tool [22]. Besides the two *D. solani* strains isolated before 2000, we sequenced two *D. solani* isolated in Switzerland during the 2005–2013 outbreak, CH05026 and CH07044. Statistics of these newly sequenced draft genomes are presented in Table 2.

### 2.3. Genome Analysis

Pairwise comparison of the genomes was computed using the average nucleotide identity (ANI) Pyani python module (https://github.com/widdowquinn/pyani [23], accessed on 21 May 2021) with the blast algorithm (ANIb). The species threshold was set at 96%.

Single nucleotide polymorphisms (SNPs) were detected with the CLC Genomics Workbench (basic variant detection tool) after mapping Illumina reads onto reference genome.

Orthologous sequences were clustered into homologous families using the SiLix software package [24], with a 70% identity threshold and at least 80% overlap. Strain-specific gene families and gene families absent in only one of the six analysed genomes were extracted from the SiLix output. Since draft genomes were analysed, the presence and position of those specific and absent genes were manually inspected to detect split and truncated genes due to their location at the end of contigs as well as genes detected on very small contigs of a few hundred nucleotides.

### 2.4. Aggressiveness Assays

Bacterial strains were plated on LB^−^ plates, incubated for 16 h at 28 °C and re-suspended in KPO_4_ 50 mM pH 7.0 buffer. After wounding with a yellow tip, potato tubers and detached chicory leaves were inoculated with 2.10^6^ bacteria and incubated at 26 °C in closed boxes to allow high humidity. After five days incubation, symptoms on potato tubers were categorised according to the following scale: 1: maceration zone <2 mm; 2: maceration zone <5 mm; 3: maceration zone <10 mm; and 4: maceration zone >10 mm. For chicory leaves, measuring the length of rotted tissue after 24 h incubation assessed disease severity. Both assays were carried out in triplicate.

## 3. Results 

### 3.1. The Swiss Collection of E. chrysanthemi

From 1985 to 2000, Agroscope Changins collected bacterial isolates mostly originating from symptomatic potato plant material sampled by Swiss organisations of certified seed potato production (VOZ, SEMAG, SGD, ASS and SGSG). The samples were gathered either by these organisations in pre-basic, basic and certified symptomatic material or by accredited field inspectors as part of the certification process. Most bacterial strains were isolated from blackleg-harbouring stems. In a few cases, they were isolated from macerated tubers; this is the case for the few strains isolated from imported certified seeds (marked NL in Table 1). All other isolates originated from plant material grown in Switzerland. Besides strains isolated from potatoes, the Swiss collection also included three strains isolated from other hosts (Table 1). These strains were identified as *E. chrysanthemi* using biochemical and ELISA immunological tests [14,15,25].

### 3.2. Diversity of the Pectinolytic Dickeya Isolated in Switzerland

**Figure 1:**
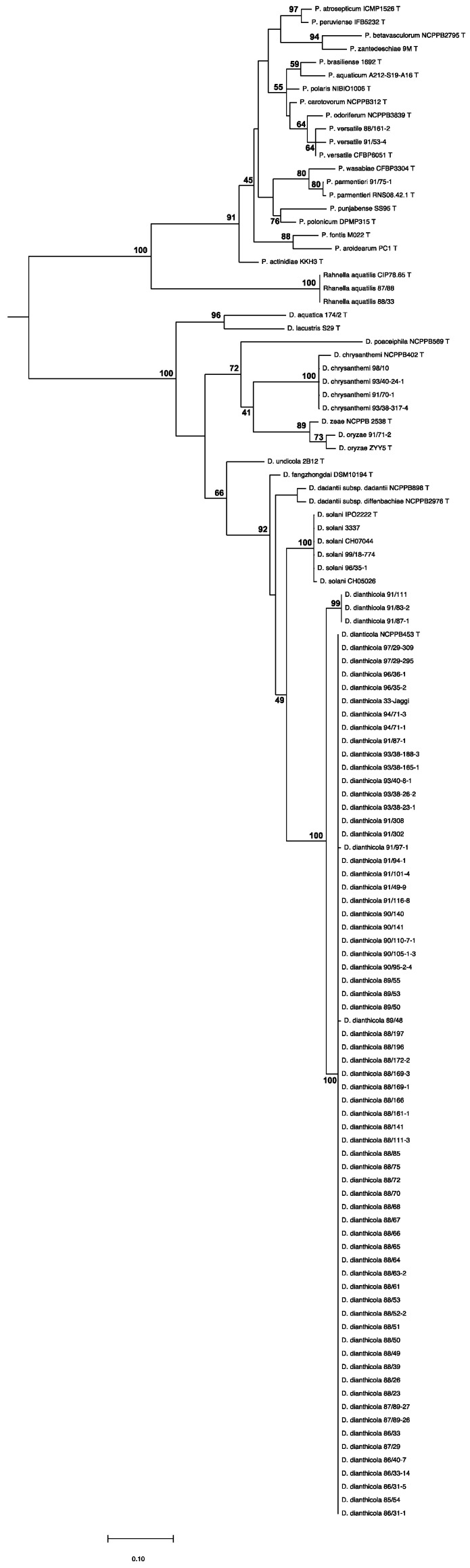
Phylogeny of strains from the Swiss collection. Phylogenic tree built up from a 799 nucleotide sequence of the housekeeping *gapA* gene using the PhyML option of the SeaView platform program (200 bootstraps).

The genus/species assignation of strains from the Swiss collection was analysed by amplification and sequencing of the *gapA* housekeeping gene recently shown to clearly differentiate the different *Dickeya* species [16] (Figure 1). Out of the 82 strains analysed, only 5 do not belong to the *Dickeya* genus, confirming the discriminative power of the biochemical and serological tests used to determine phylogenetic appurtenance before the advent of genetic tools. Three of these strains cluster with *Pectobacterium*, the other genus grouping pectinolytic bacteria commonly involved in potato blackleg disease. These include two strains belonging to *P. versatile*, a species recently described to have a very broad host range [26] and one strain belonging to *P. parmentieri*, another recently described species that was, however, present in potatoes in Poland from the 1990s [27,28]. More unexpectedly, two strains closely cluster with *Rahnella aquatilis*, a gammaproteobacterium whose genus members have been isolated from soil, freshwater and food, but also clinical samples [29]. Genomic search into the genome of the *R. aquatilis* type strain CIP 78.65 revealed the presence of genes encoding enzymes involved in pectin degradation: a pectin methyl esterase similar to PemB (52% identity/67% similarity), an exo-polygalacuronate lyase similar to PelX (54% identity/68% similarity) as well as the oligogalacturonide lyase Ogl (68% identity/82% similarity) and the various proteins involved in oligalacturonides transport and metabolism. Some *Rahnella strains* also encode a polygalacturonase showing limited similarity with *D. solani* PehN (32% identity/44% similarity in EMR1.05), albeit missing in CIP 78.65. This indicates that *Rahnella* strains indeed may possess the enzymatic arsenal to degrade pectin.

Among the 75 remaining isolates, in accordance with data from previous analyses of European *Dickeya* collections [8,9], 70 clustered with the *D. dianthicola* type strain NCPPB453. These strains are highly related since all of them but three share identical *gapA* sequences or harbour only one substitution (Figure 1). However, our analysis revealed two interesting features. The first one is the presence in the Swiss collection of four *D. chrysanthemi*—isolated in three different years, and one *D. oryzeae* strains (Figure 1 and Table 1). The second one is the occurrence of *D. solani* strains infecting potato as early as 1996, about a decade before this species was isolated in Europe [9].

### 3.3. Genomic Analysis of the Early D. solani

*D. solani* is known to be very clonal since most strains isolated either from potatoes or from ornamentals in the 2000s only harbour a few SNPs differences between them [30]. The presence of two early *D. solani* strains isolated in 1996 (CH9635-1) and 1999 (CH9918-774) in the Swiss collection (Table 1), long before this species caused the major outbreak that Europe faced later in the 2000s [7], prompted us to analyse the genomic differences that might be present as compared to epidemic *D. solani* strains isolated in the 2000s. For this, using Illumina technology, we sequenced the genomes of these two early strains as well as two other strains isolated in Switzerland after occurrence of the outbreak in Europe in 2005 (CH05026) and 2007 (CH07044). Characteristics of the draft genomes are presented in Table 2.

We compared these four genomes with the ones of the type strain IPO2222 isolated in The Netherlands [13] and of the well-described French strain 3337 [31,32]. Average nucleotide identity (ANI) analysis revealed that these six strains are highly similar with ANI percentage between them, ranging from 99.96 to 100% with a high percentage of all compared genomes being successfully aligned (99.03 to 99.99%). As already described [28], we refined this analysis by mapping the Illumina reads of the four Swiss genomes to the 3337 complete genome, allowing us to identify SNPs and InDels variations. The four Swiss strains only exhibit 53 to 94 SNPs/InDels, among which 49 are common to the four Swiss strains, indicating they are specific to the 3337 genome (Table 3). The non-common SNP/InDels are found in very few genes (Table 3). The higher number of SNPs found in strain CH07044 comes from SNPs that are clustered in one gene encoding a methyl-accepting chemotaxis protein (23 SNPs). In addition, 99.7 to 99.9% of the four Swiss genomes reads mapped to the 3337 genome, indicating that these strains do not possess inserted or extrachromosomal regions absent from 3337.

To further compare the gene content of the different *D. solani* genomes analysed here, we performed a comparative genomic analysis of the protein coding sequences of these six genomes, using the SiLix gene family clustering tool. Proteins were classified as homologous to another in a given family if the amino acid identities were above 70% on at least 80% of the full-length amino acid sequence. Out of the 4488 to 4496 protein families present in the different strains, 4272 (95.0 to 95.6%) are common to all six genomes, again pointing to the very high closeness between the strains. This was confirmed by the identification of protein families that are either present only in one strain out of the six analysed genomes (called specific) or absent in a given strains while present in the five other analysed genomes. The different strains harbour at most a few dozen specific protein families under SiLix analysis, and when screened to eliminate those resulting from sequence problems in the draft genomes (see Material and Methods), only 3 to 17 specific protein families were detected (Table 4). The vast majority of them are small (less than 100 aa) hypothetical proteins. The putative function of the remaining ones is presented in Table 4. None of these specific protein families was reported to be involved in interactions with host plants. Similarly, we detected very few protein families specifically absent in a given strain (5 to 9), and the vast majority of them are small hypothetical proteins (Table 4).

### 3.4. Aggressiveness of Early D. solani

**Figure 2:**
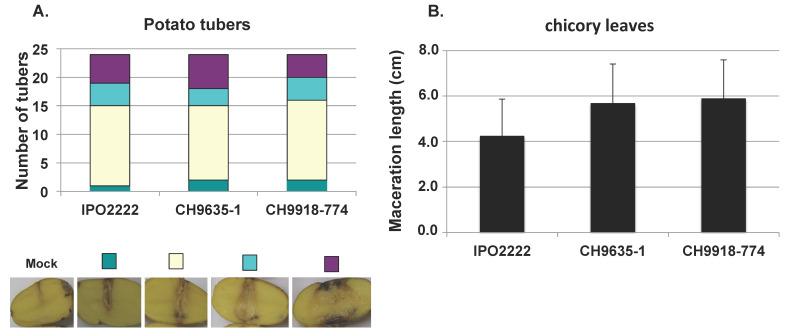
Aggressiveness of early *D. solani* strains on potato tubers and chicory leaves. For potato (**A**), symptoms were assigned to four classes according to the extent of maceration five days post inoculation (see Material and Methods). Pictures present examples of the typology of each class. For chicory leaves (**B**), disease severity was assessed by measuring the length of macerated tissue 24 h post inoculation. Assays were performed in triplicate, and the results were pooled.

Our genomic analysis revealed that the early *D. solani* possess all the genes known to be involved in virulence. However even highly closely related *D. solani* strains might vary a lot in their aggressiveness [33]. To test this, we compare the aggressiveness of the two early *D. solani* strains with that of the type strain IPO2222 on both potato tubers and chicory. On both plants, these strains were as aggressive as IPO2222 (Figure 2).

## 4. Discussion

The population dynamics of potato blackleg causing SRP is different in Switzerland as compared to in other European countries with a high incidence (60 up to 90%) of *Dickeya* species (at the time called *E. chrysanthemi*) already in the 1980s [15]. To pinpoint what may underlie this Swiss peculiarity, we analysed the diversity present in the *E. chrysanthemi* Agroscope collection gathering isolates from 1985 to 1990 and identified the majority of isolates as *D. dianthicola*, but also a few isolates as *D. chrysanthemi* and *D. oryzeae* and, interestingly, two “early” *D. solani* isolated in the 1990s from potato seeds imported from The Netherlands.

Such a high proportion of *D. dianthicola* isolates is in concordance with previous studies stating that this species was the *Dickeya* genus representative that emerged in the 1970s in potato cultures in other European countries, including those from which potato seeds are commonly imported by Switzerland [7,8,9]. The *gapA* analysis revealed a very high closeness between the Swiss *D. dianthicola* isolates. Indeed, for 67 out of 70 strains, the *gapA* sequence is identical to or only shows one SNP as compared to the NCPPB453 type strain *gapA* sequence. Only three strains harbour a slightly different sequence (23 SNP out of 799 nucleotides). This points to a high closeness between *D. dianthicola* potato pathogens that is consistent with the high homogeneity of the *D. dianthicola* genomes publicly available that show average nucleotide identities (ANI) of more than 99% between them [34]. It should be noted, however, that, except for the type strain isolated from carnation, all *D. dianthicola* genomes available are from potato isolates. It would thus be interesting to further analyse *D. dianthicola* genomic diversity by including isolates from other plants attacked by this wide host range pathogen.

Previous analyses of collections of *Dickeya* strains isolated from potato ([8,9]; 56 and 65 strains analysed, respectively) revealed the presence of only *D. dianthicola* in Europe before the occurrence of *D. solani* in the 2000s, with the exception of two *D. dadantii* strains isolated in Germany [9]. Out of the twelve *Dickeya* species described to date, five have been identified on potatoes, and they appear to be distinct on different continents [2]. Besides Europe, *D. dianthicola* was reported in Russia [2], Bangladesh [8], Pakistan [35] and Morocco [36] and has recently been determined to be responsible for a recent outbreak in the USA [37]; *D. solani* was also reported in Israel, Turkey, Russia, Brazil and Chile [13,38,39]; *D. chrysanthemi* in the USA and Taiwan [8,9,40]; *D. dadantii* in Brazil [8], Peru [9,41], Zimbabwe [42] and the USA [40]; and *D. zeae*/*D. oryzae* in Australia and Papua New Guinea [8,9,43,44]. Here, we showed that *D. chrysanthemi* and *D. oryzeae* could also be responsible for potato blackleg disease in Europe, even if much less frequently than *D. dianthicola* at the time. This broadens the spectrum of *Dickeya* species isolated from potatoes in Europe and highlights the need to not be too restrictive in the detection campaigns by only testing for the predominant species responsible for blackleg.

The other interesting finding of this study is the identification of *D. solani* strains isolated about a decade before the representatives of this species in analysed collections that were isolated during the major outbreak that spread over Europe and Israel from 2005. These early strains are highly closely related to the epidemic clones isolated later during the 2000s outbreak, harbouring very few SNP variations and differences in gene content (Table 3 and Table 4). Among these rare genomic variations, there is no linking evidence for the involvement in interactions with plants, and virulence tests revealed these early isolates to be as aggressive as the *D. solani* type strain on tubers and chicory leaves (Figure 2). Our data thus highlight the presence of potentially aggressive *D. solani* in potato seeds already in the last century, and we may wonder why it did not lead to an earlier outbreak. Though this a complex question, we may put forward a few assumptions. First, the seed lots carrying these early *D. solani* were contaminated to such levels that they were rejected for planting; if such high seed tuber symptoms were the rule for these strains, we may assume that these bacteria were not further disseminated in fields. Since the severity of diseases caused by SRP is highly dependent on temperature and humidity conditions [6,7], another possibility would be that the environmental conditions encountered by contaminated lots in further planting were not conducive enough to allow massive multiplication and spread. Finally, despite the clonal nature of *D. solani*, very few genomic differences between isolates might result in large differences in aggressiveness [33]. Even if the early *D. solani* are able to efficiently attack plants in laboratory conditions, we cannot rule out that subtle genomic changes may render these strains less efficient in colonisation or latent survival before disease expression. These aspects of the pathogen’s life cycle might be important for the comprehension of outbreak occurrence and should certainly be further investigated.

In conclusion, this work revealed that a broader than previously reported range of *Dickeya* species could be encountered on diseased potatoes in Europe, pointing to other putative actors in the population dynamics that has been observed in this crop for decades. It also reported the presence of *D. solani* in potatoes in as early as the 1990s. These early strains, such as the epidemic clones already analysed, only harboured very few genomic variations as compared to strains isolated during the large European outbreak of the beginning of this century. This further confirms the clonality of *D. solani* strains isolated from potatoes over time and, therefore, the remarkable stability of the genome of this species over more than two decades.

## Figures and Tables

**Table 1:** Bacterial strains analysed in this study.

Strain Name	Isolation Year	Origin *	Host Plant	Species Definition
*E. chrysanthemi* CH85/54	1985	CH	*S. tuberosum* cv. Ostara	*D. dianthicola*
*E. chrysanthemi* CH86/31-1	1986	CH	*S. tuberosum* cv. Désirée	*D. dianthicola*
*E. chrysanthemi* CH86/31-5	1986	CH	*S. tuberosum* cv. Désirée	*D. dianthicola*
*E. chrysanthemi* CH86/33-14	1986	CH	*S. tuberosum* cv. Bintje	*D. dianthicola*
*E. chrysanthemi* CH86/40-7	1986	CH	*S. tuberosum* cv. Désirée	*D. dianthicola*
*E. chrysanthemi* CH87/29	1987	CH	*S. tuberosum*	*D. dianthicola*
*E. chrysanthemi* CH87/88	1987	CH	*S. tuberosum* cv. Désirée	*Rhanella aquatilis*
*E. chrysanthemi* CH86/33	1986	CH	*S. tuberosum* cv. Désirée	*D. dianthicola*
*E. chrysanthemi* CH87/89-26	1987	CH	*S. tuberosum* cv. Désirée	*D. dianthicola*
*E. chrysanthemi* CH87/89-27	1987	CH	*S. tuberosum* cv. Désirée	*D. dianthicola*
*E. chrysanthemi* CH88/23	1988	CH	*S. tuberosum* cv. Désirée	*D. dianthicola*
*E. chrysanthemi* CH88/26	1988	CH	*S. tuberosum* cv. Bintje	*D. dianthicola*
*E. chrysanthemi* CH88/33	1988	CH	*S. tuberosum* cv. Désirée	*Rhanella aquatilis*
*E. chrysanthemi* CH88/39	1988	CH	*S. tuberosum* cv. Désirée	*D. dianthicola*
*E. chrysanthemi* CH88/49	1988	CH	*S. tuberosum* cv. Désirée	*D. dianthicola*
*E. chrysanthemi* CH88/50	1988	CH	*S. tuberosum* cv. Désirée	*D. dianthicola*
*E. chrysanthemi* CH88/51	1988	CH	*S. tuberosum* cv. Désirée	*D. dianthicola*
*E. chrysanthemi* CH88/52-2	1988	CH	*S. tuberosum* cv. Désirée	*D. dianthicola*
*E. chrysanthemi* CH88/53	1988	CH	*S. tuberosum* cv. Désirée	*D. dianthicola*
*E. chrysanthemi* CH88/61	1988	CH	*S. tuberosum* cv. Désirée	*D. dianthicola*
*E. chrysanthemi* CH88/63-2	1988	CH	*S. tuberosum* cv. Désirée	*D. dianthicola*
*E. chrysanthemi* CH88/64	1988	CH	*S. tuberosum* cv. Désirée	*D. dianthicola*
*E. chrysanthemi* CH88/65	1988	CH	*S. tuberosum* cv. Urgenta	*D. dianthicola*
*E. chrysanthemi* CH88/66	1988	NL	*S. tuberosum*	*D. dianthicola*
*E. chrysanthemi* CH88/67	1988	CH	*S. tuberosum*	*D. dianthicola*
*E. chrysanthemi* CH88/68	1988	CH	*S. tuberosum*	*D. dianthicola*
*E. chrysanthemi* CH88/70	1988	CH	*S. tuberosum* cv. Désirée	*D. dianthicola*
*E. chrysanthemi* CH88/72	1988	CH	*S. tuberosum* cv. Granola	*D. dianthicola*
*E. chrysanthemi* CH88/75	1988	CH	*S. tuberosum* cv. Aula	*D. dianthicola*
*E. chrysanthemi* CH88/85	1988	CH	*S. tuberosum* cv. Eba	*D. dianthicola*
*E. chrysanthemi* CH88/111-3	1988	CH	*S. tuberosum* cv. Désirée	*D. dianthicola*
*E. chrysanthemi* CH88/141	1988	CH	*S. tuberosum* cv. Désirée	*D. dianthicola*
*E. chrysanthemi* CH88/161-1	1988	CH	*S. tuberosum* cv. Désirée	*D. dianthicola*
*E. chrysanthemi* CH88/161-2	1988	CH	*S. tuberosum* cv. Désirée	*P. versatile*
*E. chrysanthemi* CH88/166	1988	CH	*S. tuberosum* cv. Désirée	*D. dianthicola*
*E. chrysanthemi* CH88/169-1	1988	CH	*S. tuberosum* cv. Désirée	*D. dianthicola*
*E. chrysanthemi* CH88/169-3	1988	CH	*S. tuberosum* cv. Désirée	*D. dianthicola*
*E. chrysanthemi* CH88/172-2	1988	CH	*S. tuberosum* cv. Désirée	*D. dianthicola*
*E. chrysanthemi* CH88/196	1988	CH	*S. tuberosum* cv. Désirée	*D. dianthicola*
*E. chrysanthemi* CH88/197	1988	CH	*S. tuberosum* cv. Désirée	*D. dianthicola*
*E. chrysanthemi* CH89/48	1989	CH	*S. tuberosum* cv. Eba	*D. dianthicola*
*E. chrysanthemi* CH89/50	1989	CH	*S. tuberosum* cv. Eba	*D. dianthicola*
*E. chrysanthemi* CH89/53	1989	CH	*S. tuberosum* cv. Urgenta	*D. dianthicola*
*E. chrysanthemi* CH89/55	1989	CH	*S. tuberosum* cv. Nicola	*D. dianthicola*
*E. chrysanthemi* CH90/95-2-4	1990	CH	*S. tuberosum* cv. Eba	*D. dianthicola*
*E. chrysanthemi* CH90/105-1-3	1990	CH	*S. tuberosum* cv. Ostara	*D. dianthicola*
*E. chrysanthemi* CH90/110-7-1	1990	CH	*S. tuberosum* cv. Bintje	*D. dianthicola*
*E. chrysanthemi* CH90/141	1990	CH	*S. tuberosum* cv. Urgenta	*D. dianthicola*
*E. chrysanthemi* CH90/140	1990	CH	*S. tuberosum* cv. Bintje	*D. dianthicola*
*E. chrysanthemi* CH91/53-4	1991	CH	*S. tuberosum* cv. Désirée	*P. versatile*
*E. chrysanthemi* CH91/70-1	1991	CH	*S. tuberosum* cv. Eba	*D. chrysanthemi*
*E. chrysanthemi* CH91/71-2	1991	CH	*S. tuberosum* cv. Eba	*D. oryzeae*
*E. chrysanthemi* CH91/75-1	1991	CH	*S. tuberosum* cv. Eba	*P. parmentieri*
*E. chrysanthemi* CH91/83-2	1991	CH	*S. tuberosum* cv. Désirée	*D. dianthicola*
*E. chrysanthemi* CH91/87-1	1991	CH	*S. tuberosum* cv. Désirée	*D. dianthicola*
*E. chrysanthemi* CH91/111	1991	CH	*S. tuberosum* cv. Urgenta	*D. dianthicola*
*E. chrysanthemi* CH91/116-8	1991	CH	*S. tuberosum* cv. Désirée	*D. dianthicola*
*E. chrysanthemi* CH91/49-9	1991	CH	*S. tuberosum* cv. Eba	*D. dianthicola*
*E. chrysanthemi* CH91/101-4	1991	CH	*S. tuberosum* cv. Désirée	*D. dianthicola*
*E. chrysanthemi* CH91/94-1	1991	CH	*S. tuberosum* cv. Eba	*D. dianthicola*
*E. chrysanthemi* CH91/97-1	1991	CH	*S. tuberosum* cv. Nicola	*D. dianthicola*
*E. chrysanthemi* CH91/302	1991	CH	*S. tuberosum* cv. Désirée	*D. dianthicola*
*E. chrysanthemi* CH91/308	1991	CH	*S. tuberosum* cv. Désirée	*D. dianthicola*
*E. chrysanthemi* CH93/38-23-1	1993	CH	*S. tuberosum* cv. Sirtema	*D. dianthicola*
*E. chrysanthemi* CH93/38-26-2	1993	CH	*S. tuberosum* cv. Sirtema	*D. dianthicola*
*E. chrysanthemi* CH93/40-8-1	1993	CH	*S. tuberosum* cv. Ostara	*D. dianthicola*
*E. chrysanthemi* CH93/38-165-1	1993	CH	*S. tuberosum* cv. Urgenta	*D. dianthicola*
*E. chrysanthemi* CH93/38-188-3	1993	CH	*S. tuberosum* cv. Désirée	*D. dianthicola*
*E. chrysanthemi* CH93/38-317-4	1993	CH	*S. tuberosum* cv. Eba	*D. chrysanthemi*
*E. chrysanthemi* CH93/40-24-1	1993	CH	*S. tuberosum* cv. Granola	*D. chrysanthemi*
*E. chrysanthemi* CH93/40-83-1	1993	CH	*S. tuberosum* cv. Bintje	*D. dianthicola*
*E. chrysanthemi* CH91/87-1	1994	CH	*S. tuberosum* cv. Désirée	*D. dianthicola*
*E. chrysanthemi* CH94/71-1	1994	CH	*Zea mays*	*D. dianthicola*
*E. chrysanthemi* CH94/71-3	1994	CH	*Zea mays*	*D. dianthicola*
*E. chrysanthemi* CH33 Jäggi	1995	CH	unknown	*D. dianthicola*
*E. chrysanthemi* CH96/35-1	1996	NL	*S. tuberosum* cv. Agria	*D. solani*
*E. chrysanthemi* CH96/35-2	1996	NL	*S. tuberosum* cv. Agria	*D. dianthicola*
*E. chrysanthemi* CH96/36-1	1996	NL	*S. tuberosum* cv. Agria	*D. dianthicola*
*E. chrysanthemi* CH97/29-295	1997	CH	S. tuberosum cv. Erntestolz	*D. dianthicola*
*E. chrysanthemi* CH97/29-309	1997	CH	*S. tuberosum* cv. Erntestolz	*D. dianthicola*
*E. chrysanthemi* CH98/10	1998	NL	*S. tuberosum* cv. Agria	*D. chrysanthemi*
*E. chrysanthemi* CH99/18-774	1999	NL	*S. tuberosum* cv. Eba	*D. solani*
CH05026	2005	CH	*S. tuberosum* cv. Agria	*D. solani*
CH07044	2007	CH	*S. tuberosum* cv. Tripla	*D. solani*
IPO2222^T^	2007	NL	*S. tuberosum*	*D. solani*
3337	2008	France	*S. tuberosum*	*D. solani*

* Abbreviations: CH: Switzerland, NL: The Netherlands.

**Table 2:** Draft genome sequences of *Dickeya solani* strains isolated in Switzerland.

Strain	Accession Number	Genome Size	Number of Contigs	Coverage	Number of CDS	Number of tRNAs
CH9635-1	GCA_016404945.1	4,872,960	52	58×	4149	51
CH9918-774	GCA_016404885.1	4,881,636	72	49×	4160	49
CH05026	GCA_016404895.1	4,874,174	52	65×	4146	54
CH07044	GCA_016404925.1	4,878,125	39	79×	4133	61

**Table 3:** SNP/InDels variations present in the Swiss strains.

Strains	CH9635-1	CH9918-774	CH05026	CH07044
# SNP/InDels	57/3	51/2	54/3	86/8
common	47/2			
remaining	10/1	4/0	7/1	39/6
# intergenic	1/1	1/0	1/1	3/3
# in tRNA	-	1	1	2
# in CDS	9/0	2/0	5/0	34/3
neutral	8	2	4	29
aa change	1	-	1	5
frame shift	-	-	-	3
# of affected CDS	4	2	5	5

**Table 4:** Specific/absent protein families in Swiss *D. solani*.

Strains	Protein Family Number	Hypothetical(*)	With Known Function
**Specific protein families**			
CH9635-1	17	16 (2)	VgrG
CH9918-774	14	9	Mobile element protein 2 truncated ABC transporter permease 2 truncated cellulose synthase CbsC
CH05026	3	3	
CH07044	8	8 (1)	
Dso3337	14	6	2 phage-related 2 truncated aconitate hydratase 2 2 truncated PotA ABC transporter 2 truncated VgrG
IPO2222	10	10 (4)	
**Absent protein families**			
CH9635-1	7	7 (2)	
CH9918-774	7	6	ABC transporter permease (truncated)
CH05026	9	9	
CH07044	8	8	
Dso3337	5	2	1 phage-related Ferredoxin PotA ABC transporter
IPO2222	8	4	2 phage-related proteins regulator YfeR (truncated) truncated CmaU-related protein

(*) Number of protein families quoted as specific because of difference in respective length exceeding 80%.

## Data Availability

The datasets generated for this study are available in Table 2.
